# Systemic inflammation-based scores and mortality for all causes in HIV-infected patients: a MASTER cohort study

**DOI:** 10.1186/s12879-017-2280-5

**Published:** 2017-03-07

**Authors:** Elena Raffetti, Francesco Donato, Salvatore Casari, Filippo Castelnuovo, Laura Sighinolfi, Alessandra Bandera, Franco Maggiolo, Nicoletta Ladisa, Massimo di Pietro, Chiara Fornabaio, Simona Digiambenedetto, Eugenia Quiros-Roldan

**Affiliations:** 1 Unit of Hygiene, Epidemiology and Public Health, Department of Medical and Surgical Specialties, Radiological Sciences and Public Health, University of Brescia, Viale Europa 11, 25123 Brescia, Italy; 2 University Division of Infectious and Tropical Diseases, University of Brescia, Brescia, Italy; 3 Hospital Division of Infectious and Tropical Diseases, Spedali Civili General Hospital, Brescia, Italy; 4 Clinical Infectious Diseases of “Azienda Ospedaliera S. Anna” of Ferrara, Ferrara, Italy; 5 Clinic of Infectious Diseases, San Gerardo de’ Tintori Hospital, Monza, Italy; 6 Clinical Infectious Diseases of “Ospedale Papa Giovanni XXIII” of Bergamo, Bergamo, Italy; 7 Clinic of Infectious Diseases, University of Bari, Bari, Italy; 8 Clinical Infectious Diseases of “Istituti Ospitalieri” of Cremona, Cremona, Italy; 9 Institute of Clinical Infectious Diseases of Catholic University of Rome, Rome, Italy

**Keywords:** Neutrophil to lymphocyte ratio, Platelet to lymphocyte ratio, Mortality for all-causes, Inflammation

## Abstract

**Background:**

Two biomarkers, the neutrophil to lymphocyte ratio (NLR) and platelet to lymphocyte ratio (PLR), have been shown to be indicative of systemic inflammation and predictive of mortality in general population. We aimed to assess the association of NLR and PLR, with risk of death in HIV-infected subjects when also taking account of HIV-related factors.

**Methods:**

We conducted a multicenter Italian cohort study from 2000 to 2012 including HIV-infected subjects naïve at antiretroviral treatment.

The associations of NLR and PLR with all-cause mortality were tested by univariate and multivariate analyses using both time independent and dependent Cox proportional hazard models. We also fitted models with a cubic-spline for PLR and NLR to evaluate the possible non-linear relationship between biomarkers values and risk of death.

**Results:**

Eight-thousand and two hundred thirty patients (73.1% males) with a mean age of 38.4 years (SD 10.1) were enrolled. During a median follow-up of 3.9 years, 539 patients died. PLR < 100 and ≥ 200, as compared to PLR of 100–200, and NLR ≥ 2, as compared to < 2, were associated with risk of death at both univariate and multivariate analyses. Using multivariate models with restricted cubic-splines, we found a linear relationship of increasing risk of death with increasing values for NRL over 1.1, and an U-shape curve for PLR, with higher mortality risk for values higher or lower than 120.

**Conclusions:**

Our data suggest that NLR and PLR can reflect the severity of the underlying systemic disturbance of the inflammatory process and coagulation leading to augmented mortality in HIV positive subjects.

**Electronic supplementary material:**

The online version of this article (doi:10.1186/s12879-017-2280-5) contains supplementary material, which is available to authorized users.

## Background

High circulating levels of markers of inflammation and coagulation, most notably C-Reactive protein (CRP), IL-6, and D-dimer, have been demonstrated to be predictors of incidence and mortality for cardiovascular (CV) and non-CV diseases in healthy subjects [1–3]. Inflammation systemic markers have been also associated with an increased risk of death from any cause in an apparently healthy adult population [4, 5].

HIV-infected people even with long-term, effective antiretroviral therapy have persistent, low grade inflammation and immune activation [6, 7], and elevated levels of inflammation biomarkers (eg. IL-6, TNF, D-dimer, fibrinogen and C-reactive protein) have been shown to be strong predictors of non-AIDS events and all-cause mortality, when also controlling for CD4 count and HIV plasma replication [7–11].

Recently, two biomarkers derived from common blood parameters, the neutrophil to lymphocyte ratio (NLR) and platelet to lymphocyte ratio (PLR), have been shown to be indicative of systemic inflammation and predictive of morbidity and mortality for both CV and non-CV diseases, mainly cancer, in HIV-negative subjects [12–14].

Different cut-off values of NLR and PLR have been used in clinical studies carried out so far, depending on subjects investigated (people with solid cancers, hematological malignancies, sepsis, cardiovascular diseases, diabetes, or the general population), types of end-point (incidence and/or prevalence of diseases, response to therapy, reactivation of diseases after treatment or death). Most studies have been performed on prognostic role of these biomarkers in patients with cancer [13, 15]. On the other hand, few studies have been carried out on these biomarkers in the general population. In the USA general population examination survey (NAHNES), the NLR average was 2.15, ranging from 2.06 to 2.44 according to ethnicity, sex, education, age, BMI, smoking, alcohol drinking, diabetes or heart conditions [16]. Also many chronic conditions are known to increase the body inflammatory status [17].

Two previous studies carried out by us showed that NLR and PLR were associated with risk of death in a large cohort of HIV-infected patients with solid cancers or lymphoma [18, 19]. However, no study has been carried out on the role of these factors as predictors of all-cause mortality in all patients with HIV infection, so far, to our knowledge.

The aim of our study was to assess the association of PLR and NLR with risk of death in HIV-infected subjects when also taking account of HIV-related factors.

## Methods

### Study population

The Italian MASTER cohort is a hospital-based multicenter, open, dynamic cohort established in the mid-1990s, with retrospective patients’ enrolment from 1986 to 1997 and prospective recruitment subsequently. Enrollment in MASTER is independent of the HIV disease stage, degree of immunosuppression or use of antiretroviral therapy. Clinical data are recorded for each patient in an electronic database every 3/4 months and data checking are performed at a central level every 6 months [20]. For the present study, we included patients enrolled in MASTER cohort from January 2000 to December 2012 before starting their first combined antiretroviral therapy (cART). Patients were subsequently follow-up during the study period, independently of undergoing cART and of time of starting therapy.

We collected data on gender, age, date of enrolment, country of origin, HIV exposure risk, viral hepatitis C or B co-infection from the MASTER electronic database. The following parameters, measured at enrolment and each year, were also retrieved: AIDS event and cancer occurrence, HIV-RNA, CD4 cell count, CD8 cell count, neutrophil, lymphocyte and platelet counts. Late presentation refers to people diagnosed with HIV with a CD4 cell count below 350/mm^3^ or with an AIDS defining event regardless of the CD4 cell count in the 6 months following HIV diagnosis. Late presentation with HIV advanced disease refers to persons diagnosed with HIV with a CD4 cell count below 200/mm^3^ or with an AIDS defining event, regardless of CD4 cell count in the 6 months following HIV diagnosis [21].

Vital status and date of death were ascertained through clinical charts, and through a record-linkage with Local Health Authority mortality registers in about one third of patients.

The study was conducted in accordance with the guidelines of the Declaration of Helsinki and the principles of Good Clinical Practice. The study protocol was approved by the local ethics committees. Written informed consent was obtained by all patients enrolled.

### Exposure factors and outcome

**Fig. 1:**
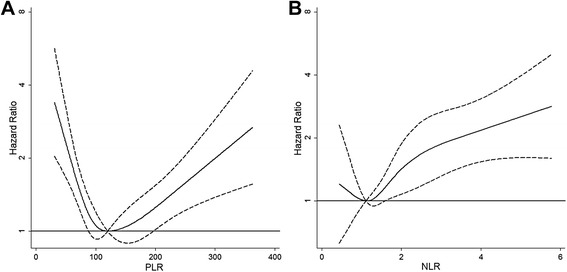
Risk of death (*Hazard Ratio*) according to distribution of PLR and NLR. **a** *PLR* was modeled by cubic spline (*solid line*) with four knots in Cox regression model adjusted for gender, age, intravenous drug use, AIDS event, CD4 cell count and antiretroviral therapy. The reference value is 120. **b** *NLR* was modeled by cubic spline (*solid line*) with four knots in Cox regression model adjusted for gender, age, intravenous drug use, AIDS event, CD4 cell count and antiretroviral therapy. The reference value is 1.1. The 95% confidence limits are shown as *dashed lines*. *Vertical axes* have a *logarithmic scale*. Abbreviations: PLR, platelet to lymphocyte ratio; NLR, neutrophil to lymphocyte ratio

The exposure factors evaluated in our study were PLR and NLR. After evaluating the variability in literature and exploring the dose-response relationship between values of these biomarkers and risk of death by spline curves in our cohort (Fig. 1), we defined three categories of biomarkers values on the basis of the shape of the spline curves, according to the following cut-offs: <100, 100–200 and ≥200 for PLR, and <2, 2–4 and ≥4 for NLR.

The primary outcome of this study was all-causes mortality.

### Statistical analysis

Follow-up was determined from date of enrollment in the cohort to 31st December 2012, or last follow-up visit, or death, whichever occurred first. Mortality rates were standardized for gender and age using the direct method with the European population as the standard and truncated at 65 years. Rates were expressed per 1000 person-years (PYs).

The associations of NLR and PLR with all-cause mortality were tested by univariate and multivariate analyses using both time independent and time dependent Cox proportional hazard models, which provided estimates of hazards ratios (HRs), their 95% confidence intervals (CIs), and *p*-values according to Wald test. In time dependent models, the study period was divided into 1 year intervals, and gender, age at enrolment, intravenous drug use as risk factors, hepatitis C or B virus co-infection and year of enrolment were included as fixed covariates, whereas AIDS events, CD4 cell count and cART were included as time-dependent covariates. On the contrary, in time independent models, we included all the covariates as detected at enrolment.

In order to compare the goodness of fit of the model with and without the inflammatory variables, we used the likelihood ratio test. Various sensitivity analyses were also performed using multivariate time dependent models in which: i) CD4 cell count was replaced with CD4/CD8 ratio, ii) cART was replaced with HIV-RNA as a dichotomous variable (positive vs negative), iii) cancer occurrence was included as a time dependent covariate, iv) inverse probability weighted technique was applied in order to adjust for losses to follow-up, v) the analysis was restricted to patients who started cART, vi) the analysis was stratified by presence of HBV (defined as presence of HBsAg) or HCV (defined as HCV ab positive) coinfection.

To evaluate whether the associations of PLR and NLR with risk of death were not linear, we also fitted time dependent Cox models with a cubic-spline for PLR and NLR, respectively [22] . We used the Akaike’s information criterion (AIC) [23] to assess fitting of models with linear and non-linear terms, and to choose the number of spline knots.

The proportional hazards assumption was investigated for each single covariate and globally by analyzing Schoenfeld residuals. We first produced the graphical plots and then carried out formal statistical tests of their independence over the rank transformation of time, but no departures from this assumption were found.

The study had more than 90% power to detect a hazard ratio of death of two for being in the highest vs the lowest category of NLR value, with a two sided test and a level of alpha of 0.05, having a ratio of about 10:1 of patients in the lowest and the highest categories of NLR, respectively.

All statistical tests were two-sided, assumed a level of significance of 0.05 and were performed using Stata software version 12.0 (StataCorp, College Station, TX, USA).

## Results

Of the 23,964 subjects enrolled in the MASTER cohort up to 2012, 13,579 were excluded because had been enrolled before 2000 and 1975 were excluded because non-naive at antiretroviral treatment at enrollment, leaving a total of 8230 subjects.

Table 1 describes the baseline characteristics of the total 8230 HIV-positive subjects (73.1% males, mean age ± standard deviation (SD) =38.4 ± 10.1). 7877 (95.7%) subjects started a cART during the follow-up with a mean time to ART start of 325.2 days. The mean (SD) CD4 cell count and HIV-RNA serum level were 395.5 (277.6) cells/mm3 and 4.6 (1.0) log_10_ copies/ml, respectively. Less than half of them had more advanced HIV infection at enrolment, with 40.7% having advanced late presentation (CD4 cell count < 200 or an AIDS-defining condition in the 6 months following HIV diagnosis). CD4/CD8 ratio mean (SD) was 0.4 (0.3) and about 40% of patients had CD4/CD8 ratio less than 0.3. Less than half had PLR between 100 and 200 and most patients had NLR less than 2 (71.8%). The NLR and PLR baseline values increased significantly with age and were significantly higher in females than males (data not shown in table). Additional file 1: Figure S1 showed the PLR and NLR distribution at enrolment: 25^th^ percentile, median and 75^th^ percentile were 83, 114 and 163 for PLR, and 1,1.4 and 2.1 for NLR. When considering the variations of NLR and PLR values during follow-up, we observed that NLR increased whereas PLR decreased overtime, mainly as a consequence of an increase of total lymphocyte count combined with a decrease of platelet count, independently of the presence of HCV co-infection (Additional file 2: Figure S2).

**Table 1:** Patients’ characteristics at baseline

Variables	Categories	Total *n* (%)
Total		8230
Gender	Male	6013 (73.1)
Age at baseline (years)	18–24	610 (7.4)
	25–34	2681 (32.6)
	35–44	3054 (37.1)
	45–54	1337 (16.3)
	≥55	548 (6.7)
	Mean (SD)	38.4 (10.1)
Country of origin	Italy	6539 (80.7)
	Others	1561 (19.3)
Year of enrolment	2000–2002	2077 (25.2)
	2003–2006	2544 (30.9)
	2007–2009	1943 (23.6)
	2010–2012	1666 (20.2)
IDU	Yes	4516 (35.8)
HBV/HCV co-infection	Yes	2430 (29.5)
CD4 cell count	0–49	523 (6.8)
	50–99	516 (6.7)
	100–199	1007 (13.0)
	200–349	1733 (22.4)
	350–499	1659 (21.5)
	≥500	2296 (29.7)
	Mean (SD)	395.5 (277.6)
CD4/CD8 ratio	<0.3	2733 (40.3)
	0.3–0.45	1356 (20.0)
	≥0.45	2689 (39.7)
	Mean (SD)	0.4 (0.3)
HIV-RNA log_10_	Mean (SD)	4.6 (1.0)
Late presentation	Yes	4516 (58.6)
Advanced late presentation	Yes	3135 (40.7)
Lymphocytes	Mean (SD)	1962.4 (896.5)
Platelets	Mean (SD)	220351.6 (80150.0)
Neutrophils	Mean (SD)	2910.9 (1456.2)
PLR	<100	2675 (39.1)
	100–200	3132 (45.7)
	≥200	1043 (15.2)
	Mean (SD)	139.1 (100.8)
NLR	<2	3826 (71.8)
	2.4	1200 (22.5)
	≥4	305 (5.7)
	Mean (SD)	1.8 (1.5)

Late presentation refers to persons diagnosed with HIV with a CD4 cell count below 350/mm3 or with an AIDS defining event regardless of the CD4 cell count in the 6 months following HIV diagnosis. Late presentation with HIV advanced disease refers to persons diagnosed with HIV with a CD4 cell count below 200/mm3 or with an AIDS defining event, regardless of CD4 cell count in the 6 months following HIV

*Abbreviations*: *SD* standard deviation, *IDU* intravenous drug use, *HBV* Hepatitis B viruses, *HCV* Hepatitis C viruses, *PLR* platelet to lymphocyte ratio, *NLR* neutrophil to lymphocyte ratio

During a median follow-up of 3.9 years (total person years = 38257.54), 539 (6.6%) patients died. Gender- and age-adjusted mortality rates decreased from 26.3/1000 PYs (19.3–33.2 CI 95%) in 2000–2002 to 14.9 (11.0–18.8) in 2003–2006, 9.9 (7.2–12.5) in 2007–2009 and 9.5 (7.3–11.7) in 2010–2012 (test for linear trend: *p* < 0.001) (data not shown).

The cumulative probability of loss to follow-up at 3 years was 20.4% (19.5–21.3%).

The prognostic role of PLR and NLR using time independent and time dependent univariate Cox regression models is shown in Table 2. PLR less than 100 and higher than 200, as compared to PLR between 100 and 200, was associated with a higher risk of death; similarly, subjects with NLR between 2 and 4 and higher than 4, as compared to less than 2, had higher risk of death in both models. These results were confirmed using both using time independent and time dependent multivariate models, including gender, age and HIV related-variables as shown in Table 3. The likelihood ratio tests for the global fit of the model, before and after addition of inflammatory variables to the full model, were significant for both NLR and PLR (*p* < 0.001).

**Table 2:** Univariate Cox proportional regression models with death for all-causes as outcome

		UnadjustedCox proportional models		Unadjustedtime-dependent Cox proportional models	
Variables	Categories	HR (95% CI)	*P* value	HR (95% CI)	*P* value
PLR	<100	1.38 (1.10–1.73)	0.006	1.21 (0.95–1.53)	NS
	100–200	Ref		Ref	
	≥200	2.27 (1.77–2.92)	<0.001	3.87 (2.97–5.05)	<0.001
NLR	<2	Ref		Ref	
	2–4	1.54 (1.19–1.99)	0.001	2.21 (1.71–2.89)	<0.001
	≥4	3.27 (2.36–4.53)	<0.001	8.06 (5.90–11.0)	<0.001

Time-dependent Cox regression models, CD8 CD4 ratio, PLR, NLR were considered at enrolment and every year. The observational period were divided into intervals of 1 year duration

*Abbreviations*: *HR* hazard ratio, *95% CI* 95% confidence interval, *PLR* platelet to lymphocyte ratio, *NLR* neutrophil to lymphocyte ratio

**Table 3:** Multivariate time independent and dependent Cox regression models

		Time independent models				Time dependent models			
		HR (95% CI)^a^	*P* value	HR (95% CI)^a^	*P* value	HR (95% CI)^a^	*P* value	HR (95% CI)^a^	*P* value
Gender	Male vs Famale	1.06 (0.81–1.37)	NS	1.25 (0.92–1.69)	NS	1.01 (0.77–1.33)	NS	1.09 (0.80–1.47)	NS
Age at enrolment (years)	<25	Ref	NS	Ref	NS	Ref		Ref	
	25–34	2.02 (0.88–4.65)	0.098	3.31 (1.04–10.58)	0.043	1.59 (0.69–3.68)	NS	1.61 (0.64–4.04)	NS
	35–44	2.56 (1.12–5.84)	0.025	4.34 (1.37–13.73)	0.013	1.94 (0.85–4.43)	NS	2.07 (0.84–5.13)	NS
	45–54	5.05 (2.20–11.59)	<0.001	8.56 (2.69–27.25)	<0.001	4.35 (1.89–9.99)	0.001	4.65 (1.87–11.58)	0.001
	≥55	6.8 (2.91–16.05)	<0.001	12.08 (3.71–39.27)	<0.001	4.93 (2.09–11.66)	<0.001	5.78 (2.25–14.84)	<0.001
IDU	Yes vs No	2.6 (2.09–3.25)	<0.001	2.46 (1.91–3.16)	<0.001	2.25 (1.77–2.86)	<0.001	2.3 (1.77–2.99)	<0.001
AIDS-event	Yes vs No					1.50 (1.09–2.06)	0.012	1.43 (1.00–2.06)	0.052
CD4 cell count, cell/mm3	0–49	6.32 (4.30–9.30)	<0.001	4.9 (3.23–7.45)	<0.001	27.46 (17.67–42.65)	<0.001	16.65 (10.26–27.02)	<0.001
	50–99	3.62 (2.41–5.43)	<0.001	2.47 (1.54–3.96)	<0.001	10.53 (6.64–16.71)	<0.001	6.19 (3.68–10.41)	<0.001
	100–199	2.55 (1.76–3.69)	<0.001	2.26 (1.50–3.40)	<0.001	5.42 (3.61–8.13)	<0.001	4.31 (2.79–6.66)	<0.001
	200–349	1.91 (1.35–2.72)	<0.001	1.8 (1.22–2.65)	0.003	3.22 (2.22–4.67)	<0.001	2.75 (1.84–4.11)	<0.001
	350–499	1.35 (0.92–1.98)	NS	1.43 (0.95–2.17)	0.090	1.57 (1.05–2.36)	0.030	1.57 (1.02–2.41)	0.040
	≥500	Ref				Ref		Ref	
PLR	<100	1.57 (1.23–1.99)	<0.001			1.47 (1.14–1.90)	0.003		
	100–200	Ref				Ref			
	≥200	1.34 (1.02–1.75)	0.034			1.42 (1.06–1.89)	0.017		
NLR	<2			Ref				Ref	
	2–4			1.17 (0.90–1.53)	NS			1.47 (1.12–1.92)	0.005
	≥4			1.82 (1.28–2.60)	0.001			2.78 (1.97–3.91)	<0.001
Antiretroviral therapy	No therapy vs cART					1.28 (1.01–1.63)	0.043	1.47 (1.13–1.91)	0.004

*Abbreviations*: *HR* hazard ratio, *95% CI* 95% confidence interval, *IDU* intravenous drug use, *PLR* platelet to lymphocyte ratio, *NLR* neutrophil to lymphocyte ratio

^a^Adjusted for all the variables in the model. Time independent Cox regression models included variables at enrolment. Time-dependent Cox regression models included gender, age at enrolment, intravenous drug use as fixed covariates, and PLR, NLR, CD4 cell count, AIDS events, antiretroviral therapy as time-dependent covariates, considering at enrolment and every year. The observational period were divided into intervals of 1-year duration

**Fig. 2:**
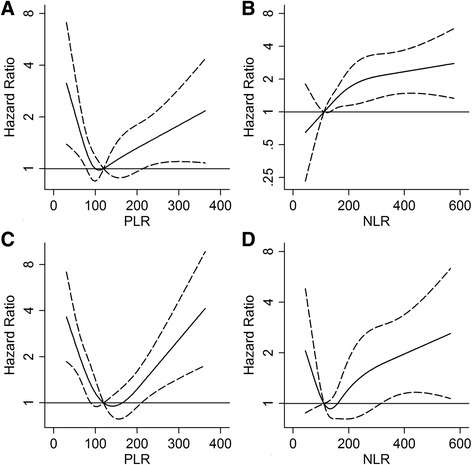
Risk of death (Hazard Ratio) according to distribution of PLR and NLR and coinfection status. (**a** and **c**) PLR was modeled by cubic spline (*solid line*) with four knots in Cox regression model adjusted for gender, age, intravenous drug use, AIDS event, CD4 cell count and antiretroviral therapy. The reference value is 120. (**b** and **d**) NLR was modeled by cubic spline (*solid line*) with four knots in Cox regression model adjusted for gender, age, intravenous drug use, AIDS event, CD4 cell count and antiretroviral therapy. The reference value is 1.1. The 95% confidence limits are shown as dashed lines. **a** and **b** included subjects without HBV/HCV coinfection, **c** and **d** included subjects with HBV/HCV coinfection. *Vertical axes* have a *logarithmic scale*. Abbreviations: PLR, platelet to lymphocyte ratio; NLR, neutrophil to lymphocyte ratio

Similar results were obtained using time dependent multivariate models with the following changes, with respect to the main analysis: i) replacing CD4 cell count with CD4/CD8 ratio, ii) replacing antiretroviral therapy with HIV-RNA positivity, iii) including cancer occurrence as covariate, and iv) weighting model for losses to follow-up (data not shown), v) limiting the analysis to patients who started a cART, vi) stratified the analysis for presence of HBV or HCV coinfection. PLR and NLR were also evaluated in multivariate time-dependent Cox regression models with restricted cubic-splines for these variables (Fig. 1), we also separated the analysis for coinfection status (Fig. 2). The risk of death increased significantly with increasing NLR over 1.1 (NLR ≥ 1.1 vs NLR < 1.1: RR = 1.80, CI 95% 1.29–2.514), whereas it was U shaped for PLR, with the lowest value at 120 and increasing risk before and after this value in whole cohort and in subjects with and without HBV/HCV coinfection.

## Discussion

This is the first study showing that increased values of NLR and PLR, two simple and reliable markers of inflammation, are associated with all-cause mortality in HIV-infected people, independently of CD4 level and other well-established HIV mortality risk factors, to our knowledge. Both NLR and PLR values measured at baseline and those measured during follow-up, analyzed as time-dependent variables, were predictive of death in these subjects.

These findings are in agreement with those from studies performed in non-HIV-infected patients, showing that inflammation is predictor of total mortality. In HIV infected people, CD4 count is considered to be the most important predictive factor of clinical outcome. CD4 count, however, was not associated with biomarkers of immune-activation or inflammation in HIV subjects under successful cART [21, 24]. In fact, recently it has been shown the potential clinical role of CD4/CD8 ratio as prognostic factor for serious non-AIDS events and death even in patients who were virologically suppressed after several years of antiretroviral therapy and CD4 recovery [25].

The NLR is believed to reflect the balance between innate (neutrophils) and adaptive (lymphocytes) immune responses, and the PLR is related with both aggregation and inflammation pathways. Theses markers have been described as predictors of either incidence of, and mortality for many diseases. Chronic low-grade inflammation measured by NLR has been found to be associated with various diseases or syndromes such as hypertension, metabolic syndrome, and osteoporosis [12, 26–28]. Furthermore, NLR was a significant prognostic factor for various diseases, including cardiovascular disease and malignancy, systemic infection or inflammatory disorders [12, 29]. Also PLR was associated with adverse cardiovascular outcome and mortality in patients with myocardial infarction [30] and with fatal outcome in subjects with various malignancies [15, 16].

However, no definite cut-offs for defining “high” and “low” values of NLR and PLR as predictors of disease incidence and mortality have been established so far. For this reason, we preferred to evaluate these biomarkers on the basis of their distribution in HIV-infected patients, and found a linear relationship of increasing risk of death with increasing values for NRL over 1.1, but an U-shape curve for PLR, with higher mortality risk for PLR values higher or lower than 120. This last finding was unexpected as no previous study found an increased risk of mortality for low values of PLR. As PLR is computed as the platelet to lymphocyte ratio, high and low values of this biomarker may be a consequence of thrombocytosis and thrombocytopenia, respectively. Both these conditions may be related with an increased risk of non-AIDS-defining events in HIV positive subjects: thrombocytosis is a well-known risk factor for vascular ischemic events, whereas thrombocytopenia has been related to high plasma levels of inflammation biomarkers such as IL-6 [31, 32], and to the risk of developing both AIDS and non-AIDS-defining events, including cancer and cardiovascular diseases [33, 34]. Accordingly, both low and high platelet count have been associated with incidence of both AIDS and non-AIDS-defining events, mainly cancer, during HIV disease [35].

HIV infection produces by itself an activation of inflammatory and coagulation pathways with elevated plasma levels of markers of inflammation and coagulation, both contributing to occurrence of serious non-AIDS events [36, 37] and to all-cause mortality even in virologically suppressed patients [9, 11]. Indeed, a recent analysis of the U.S. National Health and Nutrition Examination Survey (NHANES) data has demonstrated that a chronic, low-grade inflammation, measured as white blood cell count (WBC), is a direct, age-independent contributor to the disease burden in the general population [38]. Bastard et al. [39] have described an inflammation and immune activation status, measured with IL-6, CPR, B-2 microglobuline, D-dimer, sCD14, independently of HIV-related factors (current CD4 or CD8 values, CD4 nadir, CD4/CD8, previous AIDS event, duration or type of ART) in treated, virologically-controlled HIV-infected patients. However, discordant results of the impact of different regimens of ART on markers of inflammation have been published [40, 41].

The strengths of this study include a large population size and an unselected group of HIV-infected patients. Some limitations also exist, which should be taken into consideration when interpreting the results of this study. First, this is a multi-center observational study and therefore, although we evaluated also HIV-related factors, the potential remains for residual confounding. Second, we had no data on the causes of death, therefore we could not evaluate the associations of NLR and PLR with specific causes or groups of causes of death.

## Conclusions

Our data from a cohort of HIV-infected subjects suggest that simple indicators of systemic inflammation, as NLR and PLR, which have previously been demonstrated effective as prognostic markers in many diseases, could provide valuable prognostic information also in HIV infected patients. A comparison of these findings with other studies should be made with caution, due to substantial differences of the distribution of these biomarkers according to gender, age, ethnicity and various conditions and diseases. Our data need further validation and more studies are necessary to evaluate whether these parameters can be useful as markers of development or worsening of concomitant chronic diseases, including cardiovascular disease, kidney disease, osteoporosis and cancer, thus leading to augmented mortality in HIV patients.

## Additional files

Additional file 1: Figure S1. Distribution of PLR and NLR at enrollment. Abbreviations: PLR, platelet to lymphocyte ratio; NLR, neutrophil to lymphocyte ratio. (TIF 220 kb)
Additional file 2: Figure S2. Variations of NLR and PLR values over time, means, 95% confidence intervals and subjects at risk. Abbreviations: PLR, platelet to lymphocyte ratio; NLR, neutrophil to lymphocyte ratio; LB, lower bound; UB, upper bound. (TIF 160 kb)

## Abbreviations

AIC: Akaike’s information criterion
cART: Combined antiretroviral therapy
CI: Confidence interval
CRP: C-Reactive protein
CV: Cardiovascular
HR: Hazards ratios
NHANES: National Health and Nutrition Examination Survey
NLR: Neutrophil to lymphocyte ratio
PLR: Platelet to lymphocyte ratio
PY: Person-year
WBC: White blood cell count
