# Supplementary figures and images for: Systemic inflammation-based scores and mortality for all causes in HIV-infected patients: a MASTER cohort study
Source: BMC Infect Dis. 2017 Mar 7;17:193. doi: 10.1186/s12879-017-2280-5 (PMC5339992; doi:10.1186/s12879-017-2280-5)

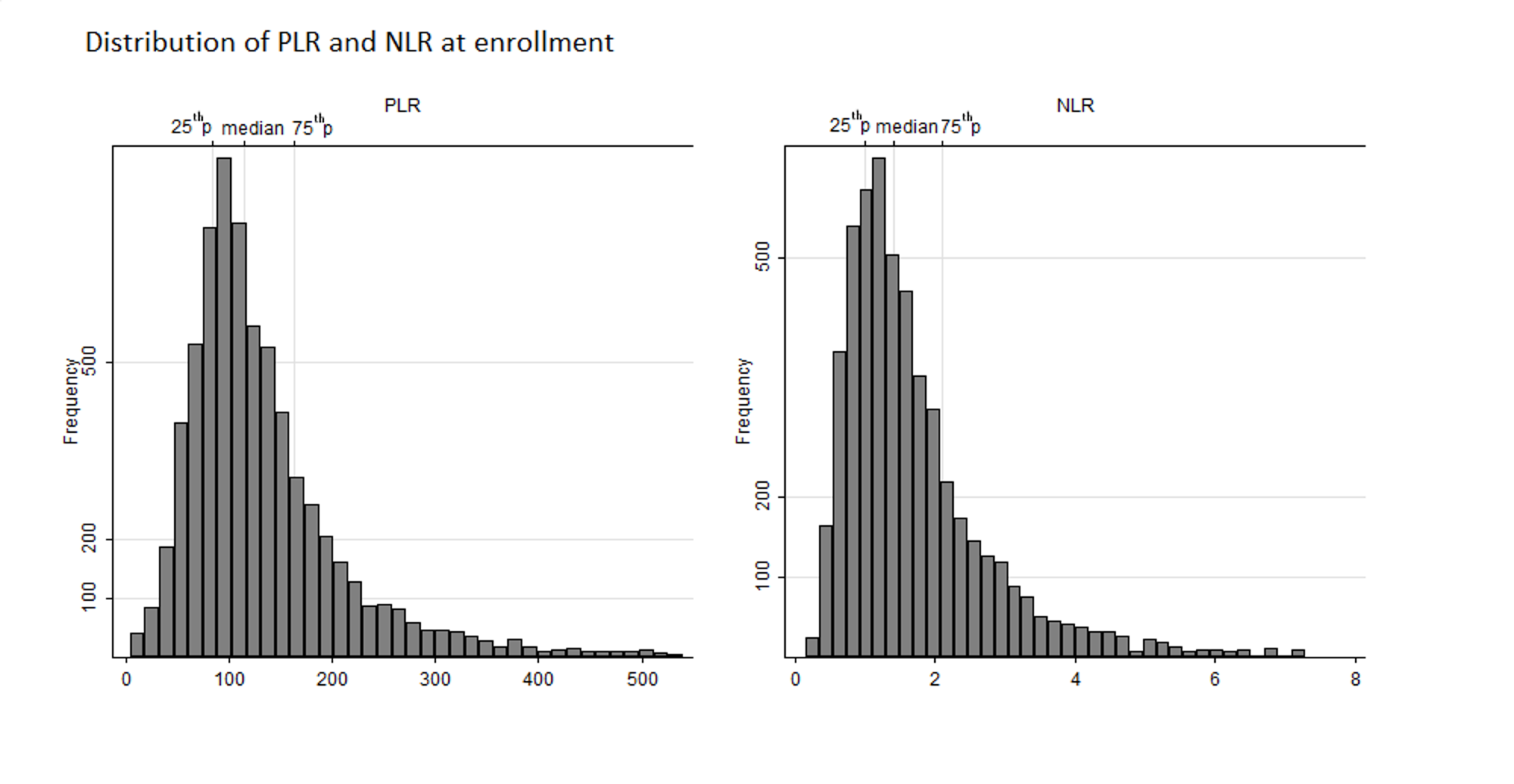

Supplement: Additional file 1: Figure S1. — Distribution of PLR and NLR at enrollment. Abbreviations: PLR, platelet to lymphocyte ratio; NLR, neutrophil to lymphocyte ratio. (TIF 220 kb) [file 12879_2017_2280_MOESM1_ESM.tif]

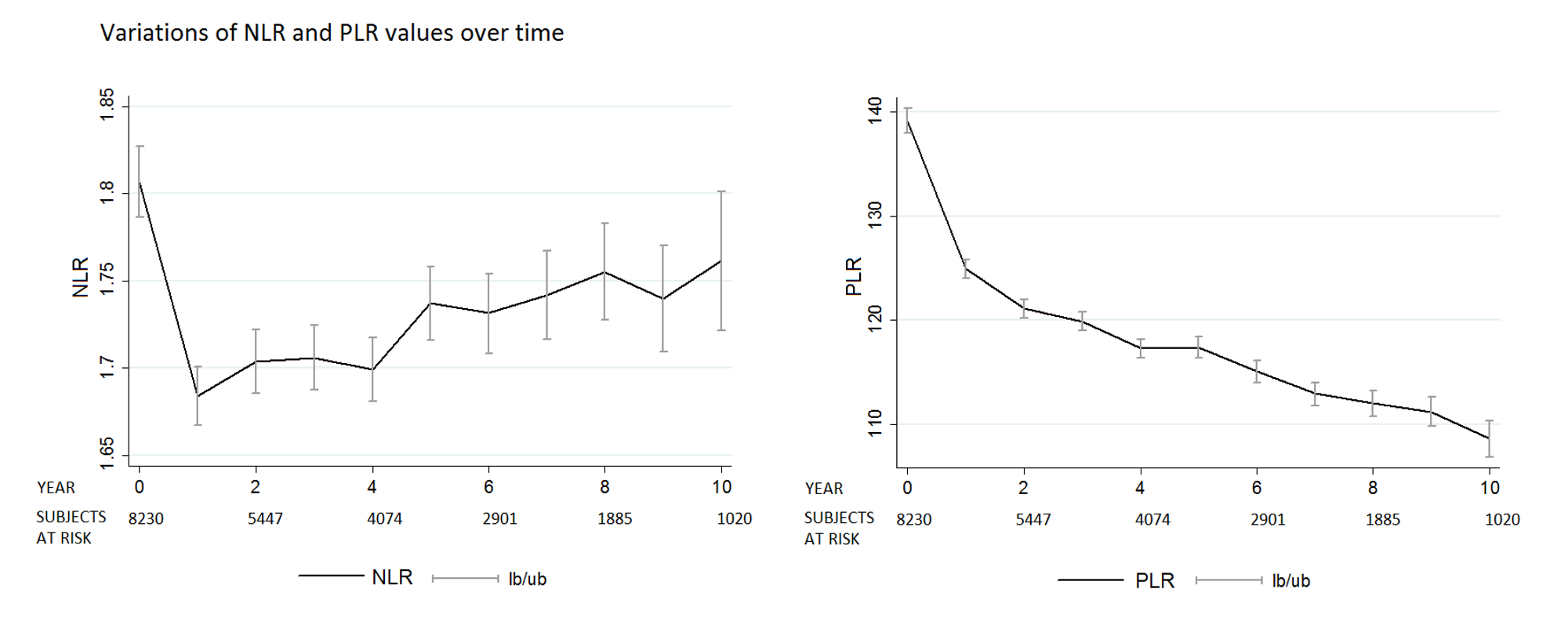

Supplement: Additional file 2: Figure S2. — Variations of NLR and PLR values over time, means, 95% confidence intervals and subjects at risk. Abbreviations: PLR, platelet to lymphocyte ratio; NLR, neutrophil to lymphocyte ratio; LB, lower bound; UB, upper bound. (TIF 160 kb) [file 12879_2017_2280_MOESM2_ESM.tif]
